# An Innovative Diagnostic Film for Structural Health Monitoring of Metallic and Composite Structures

**DOI:** 10.3390/s18072084

**Published:** 2018-06-29

**Authors:** Dimitrios G. Bekas, Zahra Sharif-Khodaei, M.H. Ferri Aliabadi

**Affiliations:** Department of Aeronautics, Imperial College London, Kensington, London SW7 2AZ, UK; z.sharif-khodaei@imperial.ac.uk (Z.S.-K.); m.h.aliabadi@imperial.ac.uk (M.H.F.A.)

**Keywords:** diagnostic film, additive manufacturing, structural health monitoring, aircraft structures, guided waves

## Abstract

A novel lightweight diagnostic film with sensors/actuators and a multiple-path wiring option using inkjet printing was developed. The diagnostic film allows for systematic, accurate, and repeatable sensor placement. Furthermore, the film is highly flexible and adaptable for placement on complex configurations. The film can be attached to the surface of the structure through a uniform secondary boundary procedure or embedded within the composite layup during curing. The surface-mounted film can simply be peeled off for repair or replacement without scratching or damaging the part. The film offers significant weight reduction compared to other available technologies. A set of extreme temperature, altitude, and vibration environment test profiles were carried out following the Radio Technical Commission for Aeronautics (RTCA) DO-160 document to assess the durability and performance of the diagnostic film for onboard application. The diagnostic film was shown to be durable and reliable in withstanding the variable operational and harsh environmental conditions of tests representing the conditions of regional aircraft.

## 1. Introduction

Structural health monitoring (SHM) based on ultrasonic techniques is now considered to provide an alternative reliable, noninvasive way to test critical structural components. In this case, SHM may involve the employment of macrofiber composite (MFC) piezoelectric actuators [1,2], fiber Bragg grating (FBG) optical fiber sensors [3,4], or piezoelectric lead zirconate titanate (PZT) transducers [5,6]. There are several challenges related to onboard SHM components that need to be addressed when dealing with large-scale structures (e.g., airframes), such as additional weight and cost of the system as well as the reliability of the decision-making. One way to address additional weight and cost is to design an SHM system with an optimal number and location of transducers while maintaining acceptable reliability in diagnosis [5,6,7,8,9]. Other challenges related to the durability, longevity, and reliability of the installation and operation of onboard equipment are reported in Radio Technical Commission for Aeronautics (RTCA) DO-160C [10]. Despite the fact that permanently attached PZT networks have proven to be a reliable tool for detecting and localizing impacts [11,12,13] and resulting damage (i.e., delamination) [14,15,16,17] in composite structures in laboratory conditions, certain issues arise when this technology is scaled up to real structures in the aviation industry [18]. More specifically, the required SHM system for monitoring the structural integrity of an aerospace structure consists of ground components, which remain on the ground, and onboard (airborne) components, which are permanently installed on the structure [19]. 

**Figure 1:**
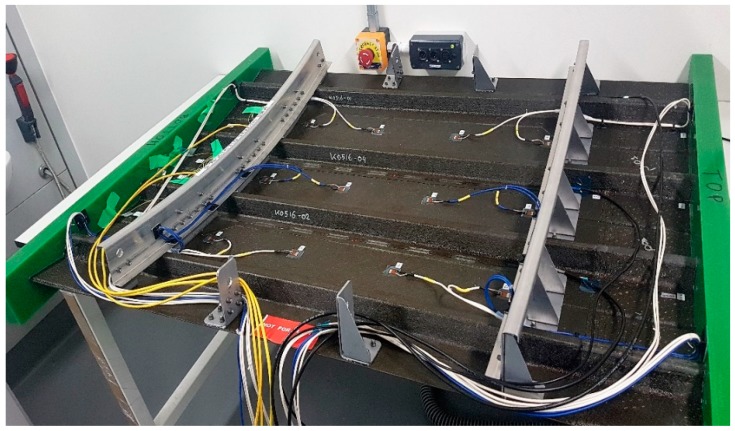
Sensorized stiffened composite curved panel.

One of the major impediments in the application of structural health monitoring using PZT-based ultrasonic-guided wave damage detection in aeronautics is the added weight, which is due mainly to the wiring. Added complications include directing and securing the wires through a complex structure such as a fuselage with frames and stringers. Figure 1 shows a sensorized stiffened composite curved panel where the challenge of additional weight can be clearly seen.

For the application of SHM systems at the industrial scale (e.g., on a fleet of aircraft), it is necessary to automate the installation. The factors that are important for scaling up the technology are precision and repeatability in placing the transducers to ensure reliability in acquisition. Another requirement is flexibility of the installation to adapt to any part with complex geometry (e.g., panels with stringers, manholes, frames) without adversely effecting its operation.

The decision to employ an SHM system as a maintenance strategy aboard a structure is directly related to the additional cost and reliability of the system as compared to existing schedule-based maintenance and SHM techniques. Furthermore, the installed sensors are usually not protected from the aircraft’s operational environment, making PZT sensor failure a distinct possibility [20,21]. In the event of a faulty transducer, if the damaged PZT cannot be replaced, it will lessen the reliability of the diagnosis, resulting in missed detections or false alarms, thereby compromising the overall performance of the system. In addition, the onboard SHM components must survive the operational and environmental loads of an aircraft without any adverse effect on their performance [19]. Hence, the reliability and probability of detection of the diagnostic system are directly related to the integrity of the onboard equipment during the lifetime of the structure and the possibility of repairing or replacing faulty components.

A possible solution to the aforementioned issues is provided by the use of additive manufacturing. Over the last few years, inkjet printing, and especially drop-on-demand (DoD) inkjet printing, has been employed as an effective and cost-efficient technique for the fabrication of electronic devices such as organic thin-film transistors [20,21], solar cells [22], organic light-emitting diodes (OLEDs) [23,24], and sensors for SHM applications [25,26,27]. Inkjet printing is a versatile noncontact printing technology that enables the creation of desirable wires on different substrates [28]. By employing this technique, the feature size of flexible electronics can be drastically reduced. In addition, the amount of material required to manufacture the circuits is significantly reduced, since the material is positioned on demand. These two key features enable a significant reduction in both the cost of producing electronic devices and their weight, while also being environmentally friendly.

**Figure 2:**
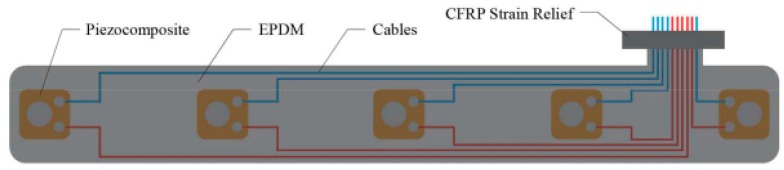
Integrated layer.

Current technologies proposed for industrial-scale use include the integrated SHM layer by [29] and the SMART Layer by Accellent [30]. The integrated layer, shown in Figure 2, consists of DuraAct^TM^ transducers integrated between two layers of uncured ethylene propylene diene monomer (EPDM) rubber. The cables for each sensor were soldered prior to their integration. The resulting layer is a flexible array that will be co-cured during the same autoclave cycle as the prepreg, which also acts as a protective layer once it is cured [14].

SMART Layer [30] is fabricated by etching a conductor pattern onto a dielectric substrate, laminating a deictic cover layer for electrical insulation, and mounting an array of PZT transducers on the circuit. SMART Layer is bonded to a metallic surface using secondary bonding with epoxy. For composite structures, the layer is embedded into the structure during the manufacturing process. The layer is reported to have been tested under harsh environmental conditions, although certain deficiencies were reported in [19].

In this paper, a flexible diagnostic film was developed to address the challenges of onboard SHM components in terms of cost, repeatability of installation, and reliability of acquisition for aeronautical application.

The proposed layer consists of an array of PZT sensors and an inkjet-printed conductive network. The diagnostic film can be either surface mounted on a structure or embedded within a composite interlayer. Due to the versatile characteristics of inkjet printing technology, the developed conductive network can be purposely designed to allow for accurate sensor placement and can better meet application demands. The reliability of the developed SHM system has been verified through extensive tests simulating the operational environment of an aircraft.

## 2. Development of the Diagnostic Film

**Figure 3:**
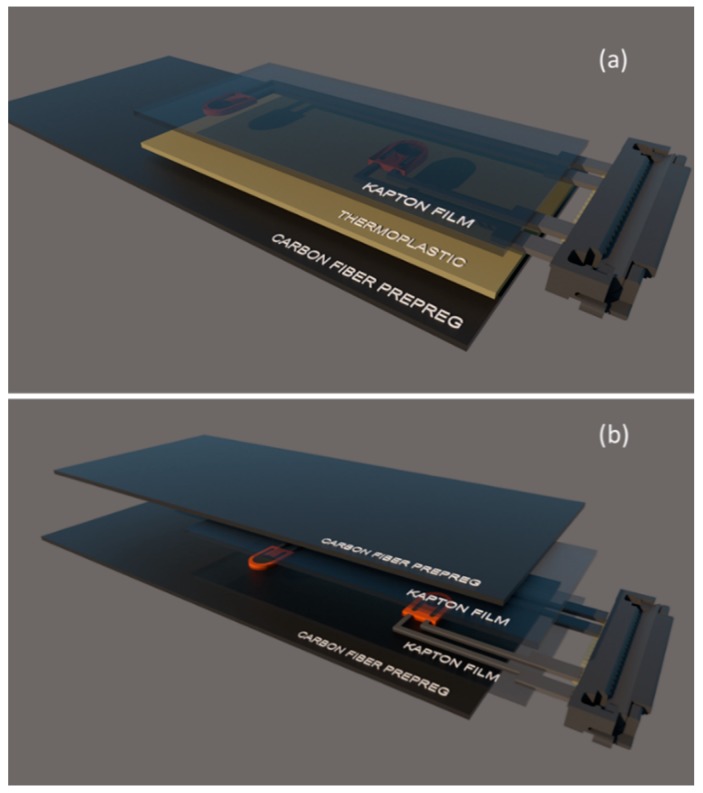
Schematic representation of (**a**) surface-mounted and (**b**) embedded diagnostic film.

A silver nanoparticle suspension in a triethylene glycol monomethyl was employed for the inkjet printing of the conductive wires. The silver nanoparticle concentration was 30–35 wt% and the particle diameter was under 50 nm. The viscosity ranged from 10 to 18 mPa·s and the surface tension was between 35 and 40 mN·m^−1^. The substrate used for printing was a 25 μm thick polyimide film (Kapton). For bonding of the Kapton film onto the composite surface, a thermoplastic film with a melting point of 150 °C was employed. Figure 3 illustrate a schematic representation of the diagnostic film surface-mounted and embedded within a composite, respectively.

DuraAct transducers were used as PZT sensors, while the connections between the printed circuits and the transducers were created using a two-part silver-loaded epoxy adhesive. A durable surface-mounted connector with an operating temperature range of −40 °C to +85 °C was also used.

**Figure 4:**
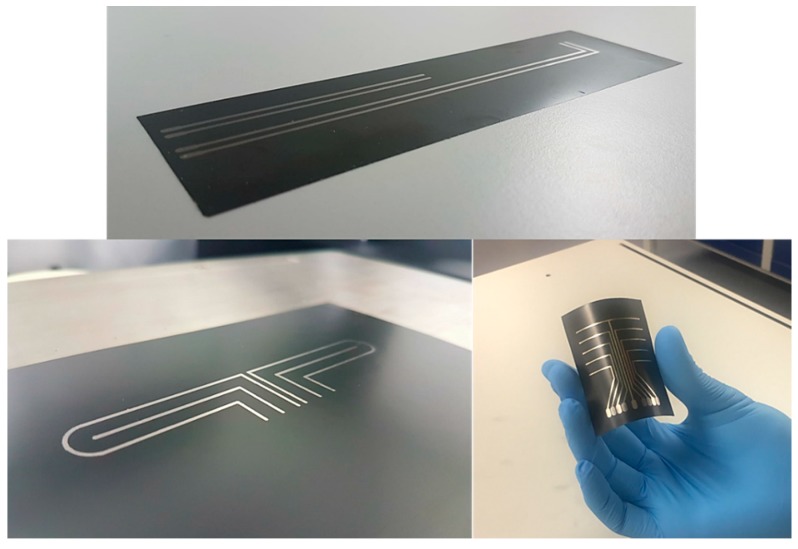
Inkjet printer and three network printed patterns.

Inkjet printing of the conductive silver wires was performed using a piezoelectric Dimatix DMP 2850 printer. The piezo voltage was selected at 20 V and a customized waveform with a maximum jetting frequency of 5 kHz enabled a satisfactory drop formation. During printing, the substrate temperature was 60 °C and a drop spacing of 30 μm was selected. The printing width was set at 1 mm. In order to decrease the electrical resistivity of the printed wires, 3 layers of silver-based ink were printed on top of each other. At the end of the printing process, sintering took place in a laboratory oven for 30 min at 150 °C to remove any remaining traces of solvents and to fuse the conductive particles into a cohesive conductive trace. The versatility of the process enables the precise and cost-effective creation of complex geometries and patterns on flexible substrates (Figure 4). Exploiting the aforementioned features of inkjet printing, specially designed circuits were developed to decrease the cross-talk between sensors and actuators. Cross-talk is an unwanted effect that greatly affects the reliability of damage detection methods, especially where conventional smart layers are employed. The use of coaxial cables lessens this effect. However, their increased weight compared to the diagnostic film is a major drawback.

### 2.1. Printed Circuit Characterization

To assess the quality of the developed film and ensure its suitability for onboard application, a set of characterization tests were carried out and are reported in the following subsections.

#### 2.1.1. Electrical Resistivity Measurements

The electrical resistivity of the inkjet-printed wires was assessed via a 4-point probe using a digital multimeter. The thickness of the printed wire was obtained from height profiles, which were measured with a surface profilometer. The electrical resistivity was calculated using:

*ρ* = R*A/*l* (1)

where *R* is resistance, and *l* and *A* are the length and cross-sectional area of the wire, respectively.

The resistivity of the printed wires was calculated using Equation (1) to 8 μΩ cm, which is approximately 5 times greater than bulk silver resistivity (1.59 μΩ cm). The calculated resistivity is slightly lower than that reported in the ink datasheet. This difference can be attributed to the additional printed layers, which increased the conduction of the paths along the printed wire. To better understand this behavior, the morphology of the printed wire surface was studied using scanning electron microscopy.

#### 2.1.2. Surface Morphology

**Figure 5:**
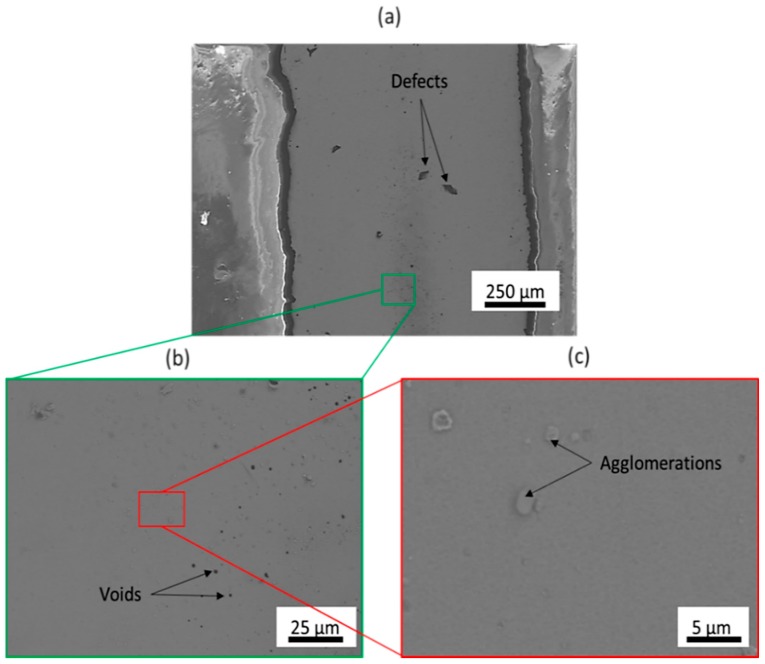
SEM images of the printed wire at the end of the sintering process. at three different magnification; (**a**): ×100, (**b**): ×1000, (**c**): ×5000.

The surface morphology of the printed wires was characterized using scanning electron microscopy (SEM). Figure 5 illustrates the SEM images obtained from the surface of a printed wire at 3 different magnifications (×100, ×1000, and ×5000) at the end of the sintering process. During the sintering process, thermal expansion of the silver particles along with evaporation of the solvent resulted in the formation of a continuous and cohesive silver layer [31]. This conductive μm-thick film provides a good percolation channel for the conduction electrons to flow. However, as can be observed in Figure 5b, there are several voids across the entire wire length that reduce the conductivity of the wire [32]. In addition, silver-particle agglomerates can be spotted in Figure 5c and can be attributed to poor dispersion quality of the silver particles within the solvent. However, it is evident that the overall surface of the printed wire consisted of a smooth and dense silver particle network that enhanced the conductivity of the printed wire. It should also be noted that the poor contrast that can be observed in the SEM image with the highest magnification can be attributed to a homogeneous particle network with a low surface roughness [33].

#### 2.1.3. Weight Assessment

As stated previously, one of the most important factors affecting the scaling up of an SHM system to real-scale applications is additional weight. Conventional ultrasonic-based SHM equipment consists of a coaxial cable and a Bayonet Neill–Concelman (BNC) connector per attached sensor. Thus, the overall weight that is added to the structure is proportional to the total number of PZT transducers, the length of the wires, the number of connectors, and, to a lesser extent, the shouldering of the sensors to the coaxial wires. The developed diagnostic film significantly decreases the overall weight of the airborne SHM system by: (i) replacing conventional coaxial cables with extremely lightweight inkjet-printed circuits, (ii) using a single surface-mounted connector instead of a number of required BNC connectors, and (iii) not having additional materials (e.g., cable clips, bundlers, protective layers) used for cable handling. It is worth noting that the weight of a single BNC connector with a 15 cm long coaxial cable is 7 g, thus the added weight of a 300 mm × 300 mm area using, for example, 4 PZT sensors, is approximately 35 g. Of course, the RAC-160 regulations are quite demanding in terms of securing the cables and connectors such that they do not become projectile during sudden descent of the aircraft. Not only are these difficulties avoided with the diagnostic film, but also the weight required for the SHM system for the same area is reduced by 67%, to approximately 12 g for the 4-transducer system.

## 3. Integrity and Reliability Assessment of the Diagnostic Film

The diagnostic film was subjected to aircraft environmental and operational loads as specified in RTCA/DO-160 for airborne components. The integrity of the film (including the transducers, wires, and bonding) was assessed by comparing the recorded response of the transducers (both actuating and sensing) before and after each test to ensure the reliability and integrity of the acquisition under repeated operational conditions. For this purpose, the developed thin film was mounted on the surface of carbon fiber reinforced polymer (CFRP) coupons using a thermoplastic layer. The diagnostic film consisted of two DuraAct sensors that were attached to the inkjet-printed wires using silver conductive paste. At the end of the printed circuits, a surface-mounted connector was employed. For the plate manufacturing, 16 plies of unidirectional Hexply 914-TS-5-134 prepreg were used. The stacking sequence was [0/45/−45/90]_2s_ and the composite final thickness was 2 mm. CFRP specimens were cut in the desired dimensions according to ASTM-STP3039 and the ends of the specimens were reinforced with end tabs. The developed thin film was tested in mechanical and electrical fatigue loading conditions. Finally, the ability of the diagnostic film to withstand thermal loading profiles required for airborne electronic components was examined.

### 3.1. Mechanical Fatigue Testing

**Figure 6:**
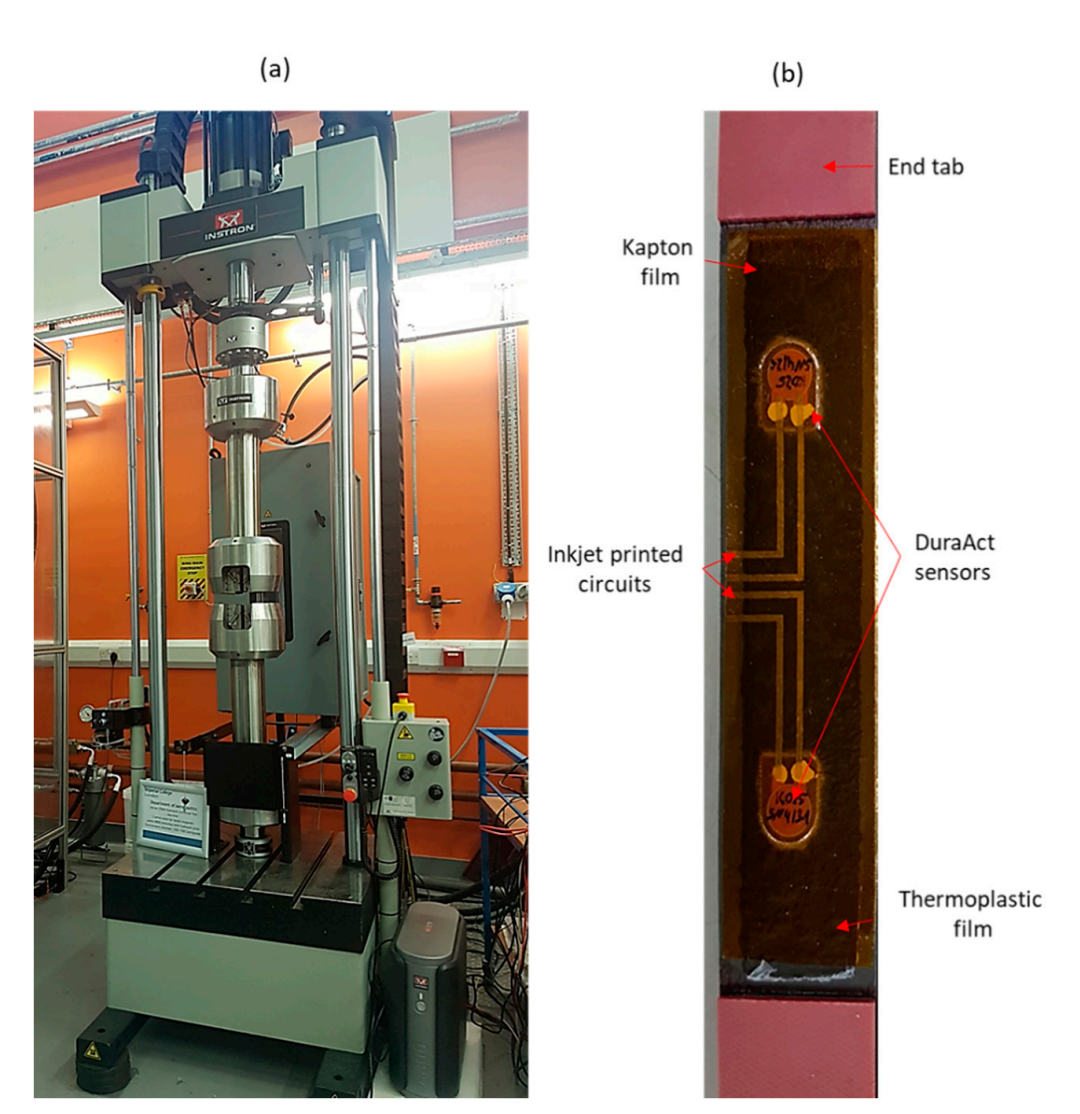
(**a**) INSTRON testing machine employed for mechanical fatigue testing of the diagnostic film; (**b**) carbon fiber reinforced polymer (CFRP) coupons with the surface-mounted diagnostic film.

In order to examine the effect of multiple fatigue cycles on the durability of the diagnostic and specifically the inkjet-printed wires, the diagnostic film was subjected to tensile-tensile fatigue testing for 10^9^ cycles, using a hydraulic INSTRON universal testing machine equipped with a 100 kN load cell (Figure 6a). The selected cyclic frequency was *f* = 5 Hz, the maximum tensile load was 5 kN, and the stress ratio was *R* = 0.1. The maximum signal amplitude of both transducers was recorded at distinctive load cycles of the test to monitor any permanent change to the signal due to mechanical loading. The CFRP with the surface-mounted diagnostic film is depicted in Figure 6b.

**Figure 7:**
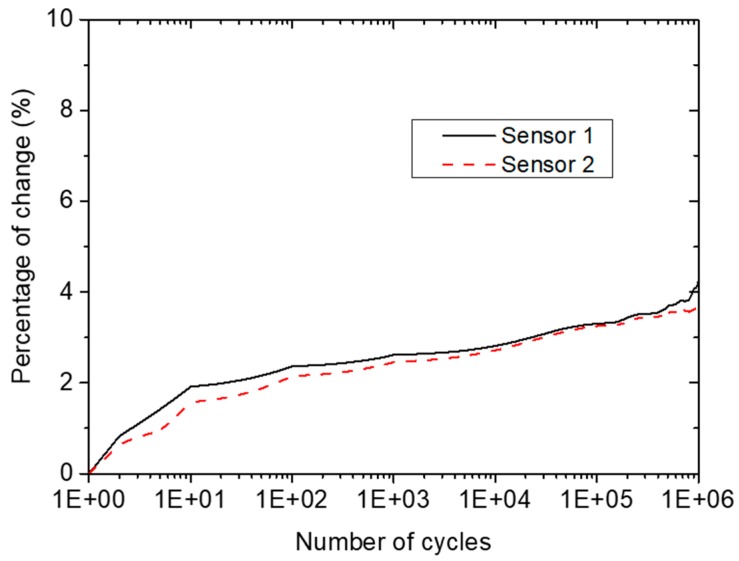
Percentage change in signal maximum amplitude versus number of fatigue cycles.

Figure 7 presents the results obtained from the fatigue testing of the coupon. The percentage change in the maximum amplitude of the propagated wave was calculated using the initial maximum amplitude as reference. As can be observed, the maximum amplitude of the signal exhibited an initial increase of approximately 2% after the first 10 cycles of mechanical fatigue load, followed by relatively stable values until the last stages of the test. This relatively low change in amplitude can be attributed to stabilization or settlement of the system during the initial loading stage. At the final stages of testing, at approximately 5 × 10^4^ cycles, the recorded signal’s maximum amplitude for both sensors showed a second change that reached almost 4%. However, it should be mentioned that the initial percentage change should not be taken into consideration due to the stabilization of the SHM system at that point. Thus, the total percentage change during the mechanical testing was approximately 2%, showing satisfactory performance for the SHM system after 10^9^ cycles of fatigue loading. This change should be considered when setting the damage threshold to avoid false alarms.

### 3.2. Electrical Fatigue Testing

**Figure 8:**
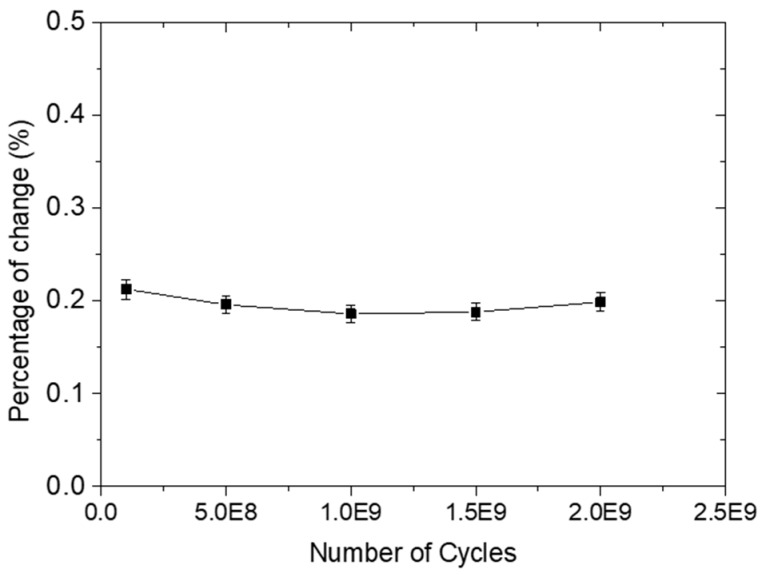
Percentage change in signal maximum amplitude versus number of electrical loading cycles.

In this case, the effect of several excitation cycles on signal amplitude was studied. For this purpose, the diagnostic film was electrically fatigued using a 250 kHz five-cycle Hanning tone-burst with an output voltage of 6 V for 0, 10^8^, 5 × 10^8^, 10^9^, and 2 × 10^9^ cycles. It should be noted that the selected voltage amplitude is extensively used for excitation of ultrasonic Lamb waves in SMH applications. At the end of the test, the maximum signal amplitude of the reference specimen was compared with those after electrical fatigue loading to see whether there was any degradation, as presented in Figure 8. It is evident that even after 2 × 10^9^ cycles of electrical fatigue loading, the maximum amplitude exhibited a minor change, less than 1%, showing the ability of the printed circuit to withstand a large number of electrical loading cycles. This behavior can be attributed to the relatively high conductivity of the printed wires, which is a result of the high-quality inkjet printing process.

### 3.3. Effect of Printed Wire Length

In a parallel study, the effect of the printed wires on both the electrical impedance and signal amplitude of the sensors was also examined. In this case, five straight wires of different lengths (50, 100, 150, 200, and 250 cm) were inkjet-printed on the Kapton surface and five DuraAct sensors were used for actuating and sensing Lamb waves. The thin film was then attached to a CFRP surface and ultrasonic waves were excited with a 250 kHz five-cycle Hanning tone-burst with a signal amplitude of 6 V. Prior to the ultrasonic measurements, electromagnetic impedance spectroscopy (EIS) was employed.

**Figure 9:**
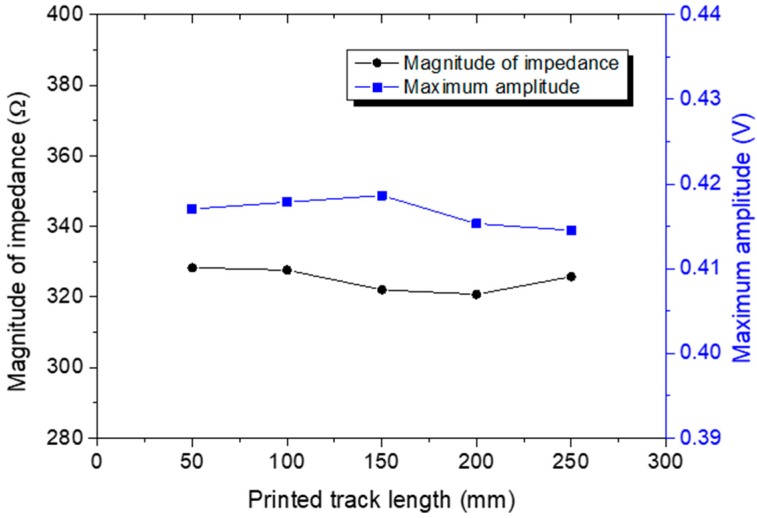
Magnitude of impedance and signal maximum amplitude versus printed wire length.

As expected, the length of the printed wires had a negligible effect on both the EIS measurements and the signal features, such as amplitude. As stated, the relatively high electrical conductivity of the inkjet-printed wires resulted in a minor attenuation of the excitation signal; see Figure 9, where the magnitude of impedance and maximum signal amplitude versus wire length are presented. Based on the obtained results, both values remained unaltered with increased wire length, indicating that the proposed diagnostic film can be employed for monitoring of large areas without compromising the performance of the SHM system.

### 3.4. Temperature and Humidity Environmental Testing

**Figure 10:**
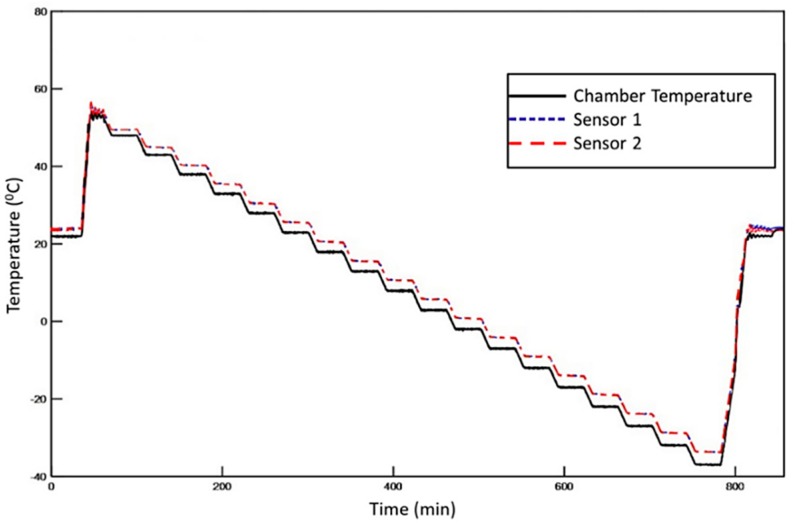
Recorded temperature profile for the chamber and two points on the composite.

Initially, the diagnostic film was mounted onto the surface of the CFRP and exposed to the environmental test profile depicted in Figure 10. Tests were performed using a TAS Series 3 temperature and climatic chamber. The chamber temperature was measured with a PRT platinum resistance thermometer probe and K-type thermocouples, which were in contact with the specimen. At the end of the thermal loading test, the effect of temperature change on electrical impedance and signal amplitude was studied. For that purpose, prior to and after the thermal loading, EIS measurements were conducted while ultrasonic signals were generated and recorded in a pitch catch configuration with excitation frequency of 50 and 250 kHz to check the effect of temperature on antisymmetric and symmetric modes. Afterward, the same procedure was followed to study the effect of increased humidity on the performance of the diagnostic film. In this case, the CFRP specimen was exposed to environmental conditions for 72 h using the climatic chamber. The relative humidity and temperature were set at 85% and 60 °C, respectively. EIS measurements and ultrasonic inspection tests were conducted prior to and after the environmental testing as described above.

**Figure 11:**
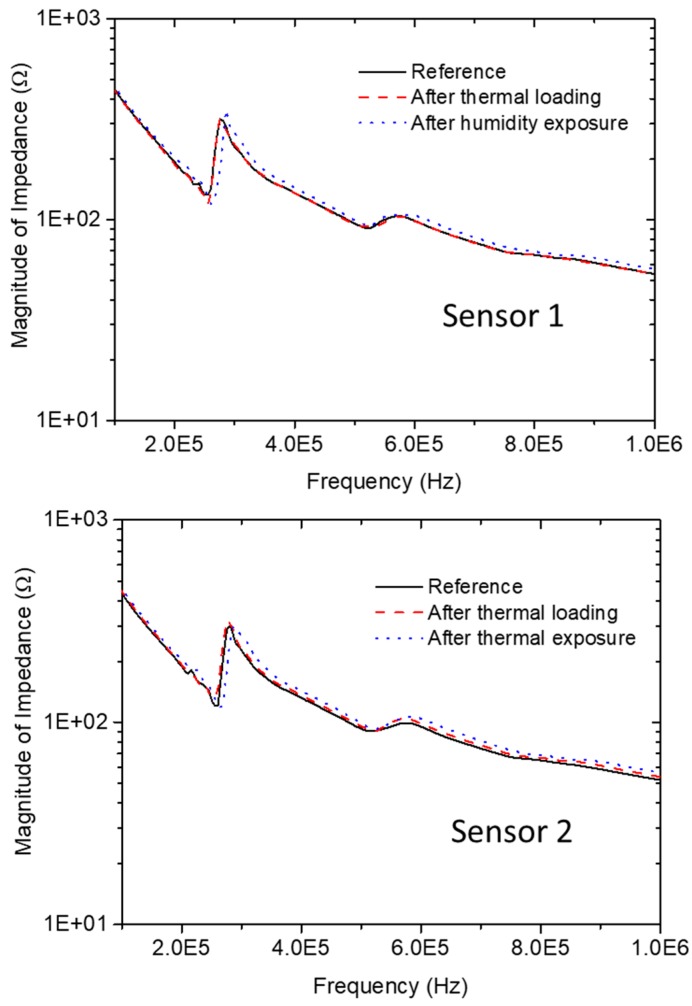
Evolution of imaginary impedance spectrum of both piezoelectric lead zirconate titanate (PZT) sensors before and after thermal loading and humidity exposure.

The evolution of the impedance magnitude spectrum for both PZT sensors before and after thermal loading and humidity exposure is presented in Figure 11. It is evident that the impedance magnitude and resonance frequencies of both sensors remained unaltered after the temperature and humidity testing. Before thermal loading, the resonance frequency and impedance magnitude at this frequency of sensor 1 were 275.72 kHz and 316.85 Ω, respectively. After thermal exposure, the resonance frequency remained the same, exactly 275.72 kHz, while the magnitude of impedance showed a minor increase to 318.49 Ω. Similar behavior was observed for sensor 2. For the reference state, the resonance frequency was found to be 278.72 kHz and the impedance magnitude at this point was 308.00 Ω. At the end of the thermal loading test, the resonance frequency was slightly shifted toward lower frequencies, reaching 276.89 kHz, while the impedance magnitude showed a minor increase to 313.85 Ω, which was within the acceptable range. As can be seen, after exposing the CFRP specimen to 85% humidity and 60 °C, the impedance magnitude and resonance frequencies of both sensors showed negligible changes.

**Figure 12:**
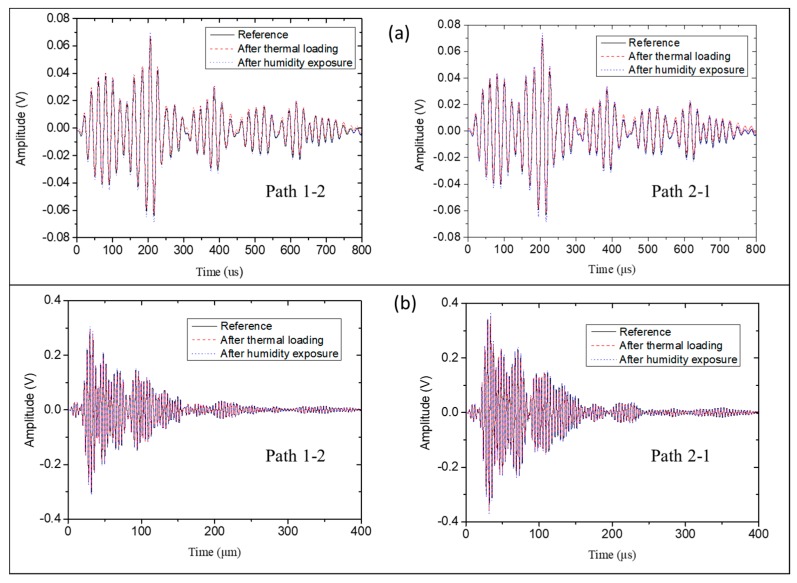
Recorded signals before and after thermal loading tests. Excitation frequencies were (**a**) 50 and (**b**) 250 kHz.

The thermal and humidity exposure of the plate to the operational temperature profile did not affect the recorded signals. For both excitation frequencies (50 and 250 kHz), the features of the recorded signals showed minor changes, indicating that the developed thin film acted as a protective layer for both the PZT transducers and the inkjet-printed circuits (see Figure 12).

## 4. Diagnostic Film Applications

**Figure 13:**
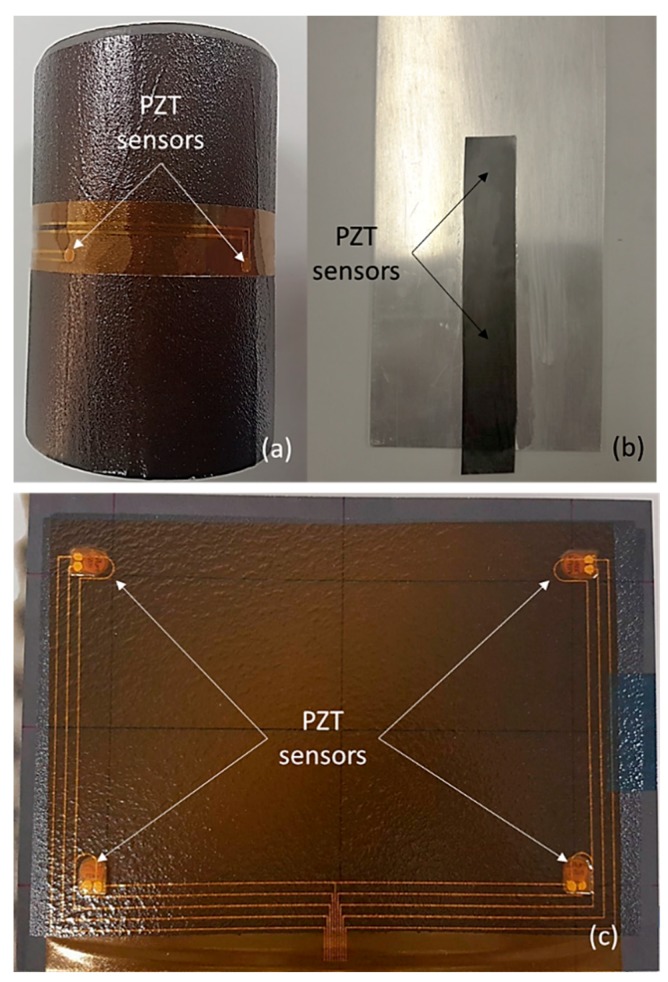
The developed thin film attached to (**a**) cylindrical CFRP, (**b**) aluminum, and (**c**) CFRP plate.

In this section, examples of embedding or surface-mounting of the developed diagnostic film are presented. Figure 13 depicts three cases where the thin film was attached on the surface of a metallic or composite plate using different PZT sensors. In detail, Figure 13a shows the application of the thin film attached to a curved surface, a cylindrical CFRP composite tube. The employed PZTs in this example were two 0.5 mm thick transducers by PI Ceramic. The diagnostic film can also be surface-mounted on an aluminum plate (Figure 13b). By using the thermoplastic film, good adhesion was achieved, while any short circuit between the metallic plate and the printed electrodes was successfully avoided. For the last case scenario, depicted in Figure 13c, the film was mounted onto the surface of a CFRP plate. Four DuraAct sensors were placed in the corners of the 225 mm × 300 mm panel, showing the film’s flexibility to adapt to large geometries of complex shape. The dashed lines in Figure 13c indicate that the cables were printed on the back surface of the diagnostic film.

**Figure 14:**
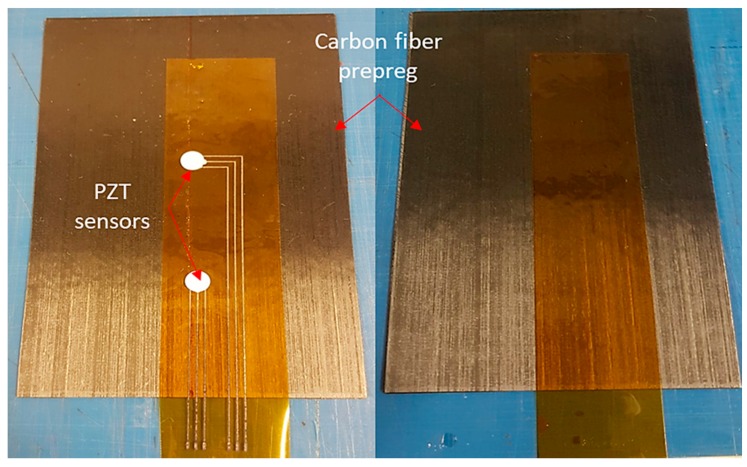
Thin film embedded within a CFRP interlayer.

The diagnostic film can also be embedded into composite. Figure 14 depicts the proof of concept for embedding the thin film within a composite interlayer. To avoid short circuiting between the conductive printed circuit and the carbon fibers, the wires and sensors were sandwiched using a second Kapton film (Figure 14). To improve adhesion between the two Kapton films and avoid possible delamination, a chemically treaded Kapton film was used as an insulation layer. Afterward, curing of the carbon fiber prepreg with the embedded network of PZT sensors took place in autoclave.

**Figure 15:**
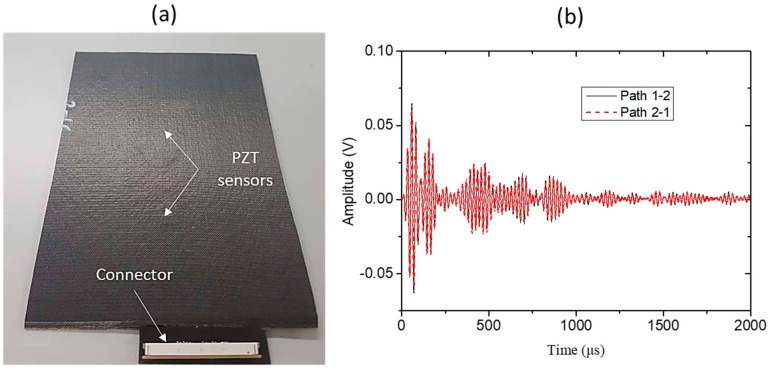
(**a**) Diagnostic thin film embedded within a CFPP plate and (**b**) the recorded signals from the PZT sensors.

At the end of the curing process (Figure 15a), the connector was surface-mounted on the Kapton film and signals were recorded to examine the system’s ability to withstand the increased temperature and pressure inside the autoclave. As can be seen in Figure 15b, the integrity of the sensors and the connections between the printed circuits and the PZTs was confirmed by the successful recording of the signals.

## 5. Conclusions

The design, manufacturing, and testing of a novel flexible diagnostic film for SHM application to aeronautical structures was presented. The diagnostic film has the following novel characteristics:

- Is flexible, so that it can adapt to any surface.
- Can be surface-mounted as well as embedded in composites.
- Is repairable, so it can be easily removed from the surface of the structure and replaced in case of failed sensors.
- Has an adaptable design: the wires can be of any shape (curved or straight), so it is applicable to any complex geometries and can be readily scaled up.
- Has low weight (compared to conventional wired solution, 70% reduction in weight can be achieved).
- Has a uniform and repeatable bonding quality.
- Exhibits reliable functionality under operational conditions of aircraft.

The integrity and reliability of the signal acquisition was assessed under the operational conditions of an aircraft. For that purpose, the developed diagnostic film was subjected to extensive testing that included electrical and mechanical fatigue and thermal loading. The data acquired before and after each test showed successful application of the diagnostic film under loading conditions representative of an aircraft, without any permanent change. The collected data can then be reliably used for any diagnostic application, including impact and/or damage detection and identification of aeronautical structures.
